# Quantitative assessment of fracture healing using patient-specific CT finite element analysis: A novel approach to external fixation management

**DOI:** 10.1016/j.tcr.2025.101245

**Published:** 2025-08-22

**Authors:** Yusuke Matsuura, Takahiro Yamazaki, Seiji Ohtori

**Affiliations:** a Department of Orthopaedic Surgery, Graduate School of Medicine, Chiba University, 1-8-1 Inohana, Chuou-ku, Chiba city, Chiba, Japan

**Keywords:** Finite element analysis, Callus formation, Bone union, Bone strength, Tibia fracture, External fixation

## Abstract

**Background:**

Posttreatment decisions regarding fracture care primarily rely on physicians' subjective judgment based on clinical and imaging findings. This study aimed to evaluate whether patient-specific computed tomography (CT) finite element analysis could allow for the quantitative assessment of fracture site strength as an objective indicator of treatment decisions.

**Methods:**

Our patient was a 37-year-old male who underwent external fixation for an open comminuted tibial fracture of the right lower leg. Monthly CT scans were taken starting 2 months postinjury. Patient-specific CT finite element analysis was used to evaluate weight-bearing capacity both with external fixation and after its removal. Treatment decisions were based on the analysis of the minimum principal strain and callus failure risk in the callus region.

**Results:**

For the first 3 months postfixation, destructive strain occurred in the callus even with the external fixator, indicating a risk for nonunion with full weight bearing. At 4 months postsurgery, the mechanical stability of the callus improved, indicating potential bone union under daily activity loads. At 5 months, CT images showed partial callus bridging in the posterior tibia, with finite element analysis demonstrating its capacity for withstanding loads of up to 1724 N after external fixator removal. These results suggest the need for careful management of weight-bearing load early after external fixation.

**Conclusion:**

This study demonstrated the possibility of implementing postoperative weight-bearing management among patients with external fixation based on quantitative assessment using patient-specific CT finite element analysis rather than relying solely on clinical experience. Further accumulation of clinical data is expected to improve analytical accuracy and contribute to the establishment of fracture treatments based on more objective indicators.

**Clinical relevance:**

This study demonstrated the potential clinical application of patient-specific CT finite element analysis in fracture treatment.

## Introduction

Fractures are extremely common injuries that are crucial for physicians involved in trauma care. Generally, fractures with minimal displacement are treated conservatively with external fixation, whereas those with significant displacement or unstable fracture sites require surgical intervention followed by early range of motion exercises. However, determining the optimal timing for various clinical decisions, such as when bone union has occurred, when to remove external fixation, when to permit weight-bearing, and when to allow return to work or sports activities, is often significantly challenging for clinicians.

This complexity in decision-making stems from the need to consider multiple factors, namely patient-specific factors (e.g., body weight and bone density), fracture characteristics (e.g., the degree of comminution), and treatment variables (e.g., implant rigidity and final fixation method), as well as the lack of objective criteria for evaluating fracture site stability under external forces. Indeed, it would be perfectly reasonable to state that the treatment of individual fracture cases is currently largely dependent on the treating physician's subjective judgment based on their clinical experience.

Bone union requires two essential elements: biological activity and physical stability. Regarding physical stability, Perren reported that strain levels between 2 % and 10 % at the fracture site could promote favorable callus formation and subsequent bone union [1]. Therefore, we hypothesized that establishing a method that could measure and evaluate strain at each patient's fracture sites would enable the objective assessment of appropriate surgical fixation methods and postoperative rehabilitation protocols.

Patient-specific CT-based finite element analysis (FEA) offers an objective method for evaluating the mechanical behavior of the bone. This technique enables computer simulation of three-dimensional bone models that reflect the patients' bone density distributions, allowing the measurement of mechanical behavior in conjunction with implants. Multiple validation studies using fresh-frozen cadavers have established the reliability of patient-specific CT-based FEA [[2], [3], [4]]. Furthermore, the ability of patient-specific FEA to analyze callus formation during the bone healing process has been validated using rabbit femoral defect models [5]. We propose that applying these techniques in clinical settings could enable the evaluation of fracture site stability for individual patients, potentially informing rehabilitation protocol decisions.

The current study therefore aimed to investigate whether patient-specific CT-based FEA could be used to determine appropriate weight-bearing loads and optimal timing for external fixator removal in patients treated with external fixation for right tibial shaft fractures. This investigation was accomplished by conducting sequential measurements to determine the allowable weight-bearing load necessary for bone union.

## Materials and methods

### Case description

**Fig. 1:**
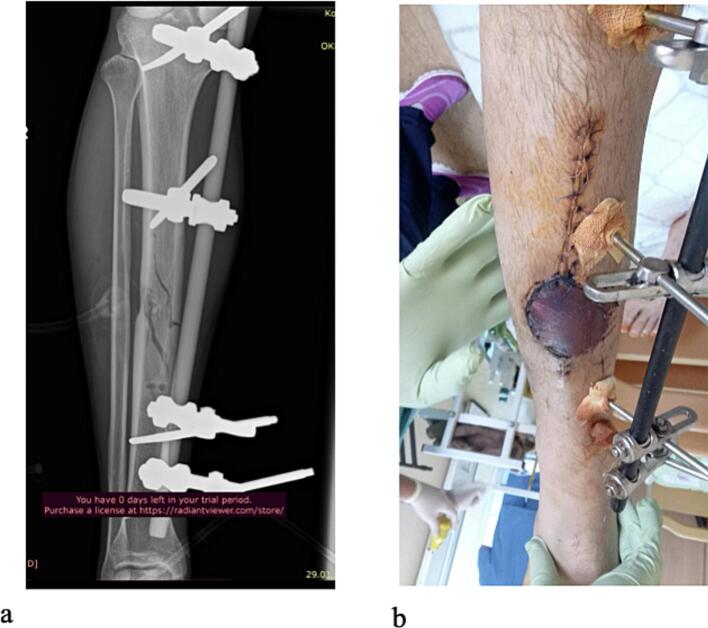
Clinical information from the previous hospital. a. Plain radiographs. b. Postoperative image after the flap surgery.

A 37-year-old male military medic sustained injuries from a mortar fire while on active duty. The mechanism was classified as secondary blast injury, with explosion-propelled fragments causing a comminuted tibial fracture with a soft tissue defect in the right lower leg and a distal triceps surae muscle defect in the left lower leg. The patient initially underwent external fixation and negative pressure wound therapy (NPWT), followed by a veno-accompanying artery fascio-cutaneous flap on his right lower leg 1 week after the injury (Fig. 1). The patient was then referred to our hospital 2 months postinjury for continued treatment.

**Fig. 2:**
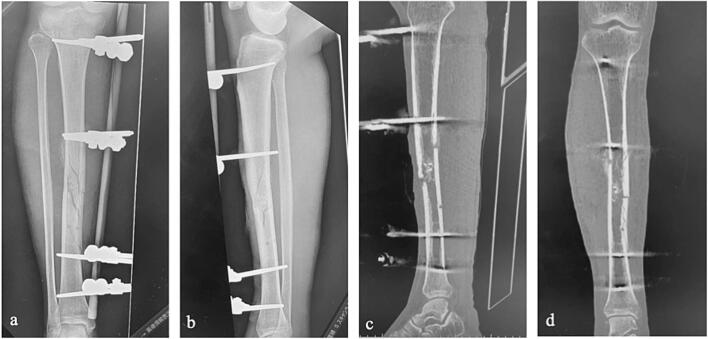
Radiological findings upon initial presentation. a. Anteroposterior plain radiograph. b. Lateral plain radiograph. c. Sagittal computed tomography image. d. Coronal computed tomography image.

**Fig. 3:**
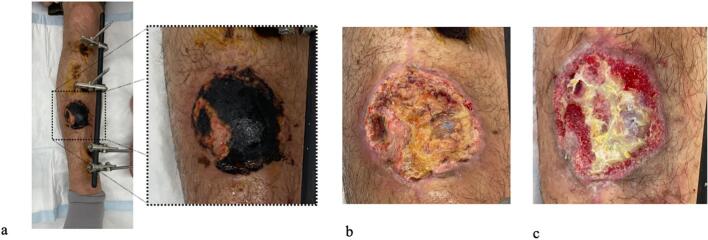
Progressive changes in the anterior flap over the fracture site. a. Anterior view of the lower leg upon initial presentation (2 months after injury) showing necrosis of the superficial flap. b. After debridement of necrotic tissue, bone exposure was avoided. c. One month after NPWT application showing areas with poor granulation tissue formation.

**Fig. 4:**
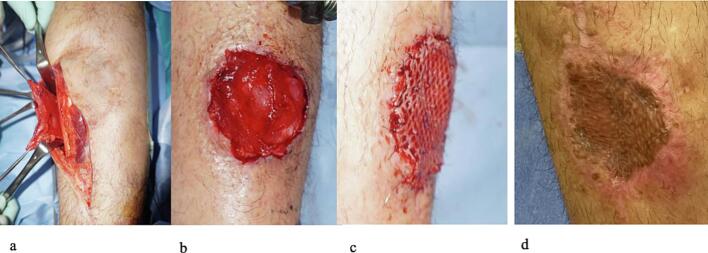
Fasciocutaneous flap reconstruction. a. Elevation of the anterior fasciocutaneous flap of the lower leg. b. Coverage with the fasciocutaneous flap. c. Coverage with split-thickness skin graft. d. Final skin condition.

Initial examination at our facility revealed a right tibial fracture stabilized with external fixation, showing excellent reduction. Radiographic examination revealed early callus formation (Fig. 2). The anterior flap over the fracture site was necrotic, with the bone remaining barely covered after debridement (Fig. 3a and b). Despite 1 month of NPWT using RENASYS TOUCH (Smith & Nephew, Watford, UK), the central portion showed no epithelialization, and multidrug-resistant *Acinetobacter* sp. (MDRA) was isolated from the wound (Fig. 3c). At 3 months postinjury, fasciocutaneous flap reconstruction with split-thickness skin grafting was performed following adequate debridement (Fig. 4a–c), which achieved complete wound closure by 4 months postinjury (Fig. 4d). The left triceps surae defect was reconstructed using the semitendinosus tendon for Achilles tendon reconstruction at 3 months postinjury, which required a 2-month nonweight-bearing period.

Given the mandatory 2-month nonweight-bearing period for the left lower extremity, treatment options for the right tibial fracture included conversion to intramedullary nailing or plate fixation. However, considering the excellent initial reduction, existing callus formation, compromised blood supply in the anterior flap region, MDRA infection, and the patient's preference, we elected to continue with external fixation to achieve bone union.

This study, which prospectively evaluated fracture site strength in patients undergoing fracture treatment using patient-specific CT-based FEA, was approved by our hospital's Institutional Review Board. Informed consent was obtained from the patient prior to participation.

### CT

CT scans of the entire lower leg with external fixation were performed monthly starting 2 months after surgery until bone union using a calibration phantom (QRM-BDC, QRM, Möhrendorf, Germany). The scans were performed using an Aquilion ONE CT system (Canon Medical Systems Inc., Tochigi, Japan) with a slice thickness of 0.5 mm and a pixel size of 0.45 mm. CT DICOM data were analyzed by importing them into Mechanical Finder version 12 (Research Center of Computational Mechanics Inc., Tokyo, Japan), a FEA software.

### Model creation

**Fig. 5:**
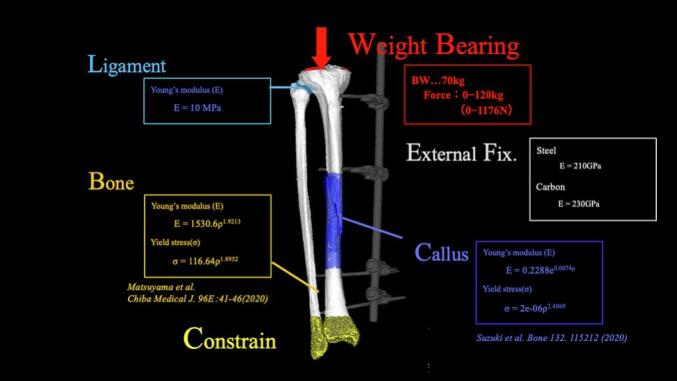
Material properties and boundary conditions.

The tibia and fibula were constructed using 2-mm tetrahedral elements, with 0.3-mm mesh elements created on their surfaces. The fracture gap was defined as the callus region and was filled with 1-mm tetrahedral elements. The proximal tibiofibular ligament was modeled using 1-mm tetrahedral elements between the proximal tibia and fibula to stabilize the tibiofibular relationship. Based on CT morphological data, the external fixator was modeled using 1-mm tetrahedral elements. Consequently, two models were created, namely an external fixation model including the fixator and a removal model with the fixator computationally removed (Fig. 5).

Young's modulus and yield stress for the bone and callus were calculated using the following equations based on previous studies [2,5]:

(1) $$E_{\text{Bone}}=1530.{\text{6ρ}}^{1.9213},\sigma_{\text{Bone}}=116.64\rho^{1.8952}$$

(2) $$E_{\text{Culus}}=0.2391e^{8.00\rho },\sigma_{\text{Culus}}=30.49\rho^{2.41}.$$

Young's modulus of the proximal tibiofibular ligament was set at E = 10 MPa. Poisson's ratios were set at 0.3 for both the bone and callus and 0.49 for the ligament. For the external fixator materials, stainless steel and carbon were assigned Young's moduli of E = 210 and 230 GPa, respectively, with Poisson's ratios of 0.34 and 0.167, and were considered nonfailing materials. The distal portions of both the tibia and fibula were constrained, and considering the patient's body weight of 70 kg, axial loads were applied to the proximal tibial articular surface in 10-N increments until sufficient failure occurred (Fig. 5).

### Evaluation parameters

After visualizing the overall deformation and minimum principal strain in the callus region, we measured the compressive plastic risk that occurred in the callus. Given that elements with compressive plastic risk exceeding 100 % were considered as callus failure, the callus destruction ratio was defined as the volume of regions where the compressive plastic risk exceeded 100 % divided by the total callus volume. This parameter was evaluated chronologically at different postoperative time points.

## Results

### Computational requirements and analysis time

Table 1 summarizes the average number of nodes, elements, and computational time for the models. The mean computational times (h) for the external fixation model and removal model were 10.0 ± 2.62 and 6.13 ± 1.04, respectively. Including the model construction time, all results were obtained within 24 h from the CT data acquisition.

**Table 1:** Average number of elements and average computation time for each model.

	Grid points	Solid elements	Shell elements	Computational times (h)
External fixation model	714,521 ± 74,359	3,626,903 ± 374,990	191,604 ± 1498	10.0 ± 2.62
Removal model	357,867 ± 39,874	1,837,276 ± 175,847	194,938 ± 6790	6.13 ± 1.04

### External fixation model (Fig. 6)

We analyzed the feasibility of walking with external fixation. At 2 months, large strains were observed in the callus region, with a rapid increase in callus failure rates recorded at a of load of approximately 627 N. Similarly, at 3 months, a sudden increase in callus failure rates was observed at a load of approximately 804 N. In contrast, at 4 months, minimal principal strains below −0.1 rarely occurred within the callus region, and no sudden increase in callus failure rates was observed. Based on these results, we decided to limit weight bearing to 1/2 PWB (partial weight bearing) while the external fixator was in place due to concerns about callus failure under full weight-bearing loads even with external fixation during the first 3 months after surgery. However, at 4 months, the risk of callus failure was low, which allowed for full weight-bearing and unrestricted activities of daily living.

**Fig. 6:**
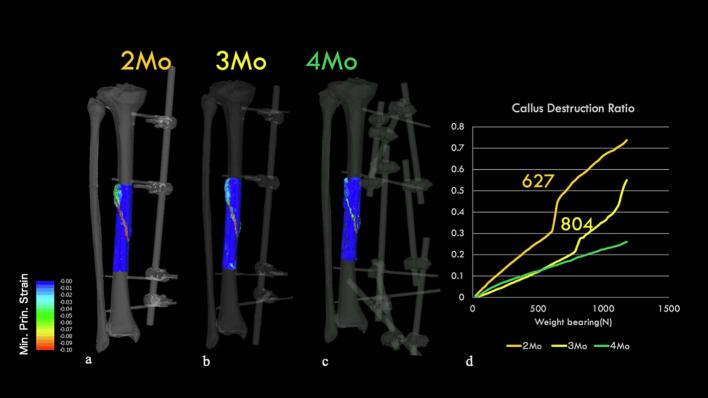
External fixation model: Minimum principal strain contours under 1000 N load. a. At 2 months: Most areas of the callus showing minimum principal strain below −0.1. b. At 3 months: A decrease in the areas showing minimum principal strain below −0.1. c. At 4 months: Almost complete disappearance of areas with minimum principal strain below −0.1. d. Callus failure rate: At 2 and 3 months, we observed a load threshold at which a rapid increase in callus failure occurs.

### Removal model

**Fig. 7:**
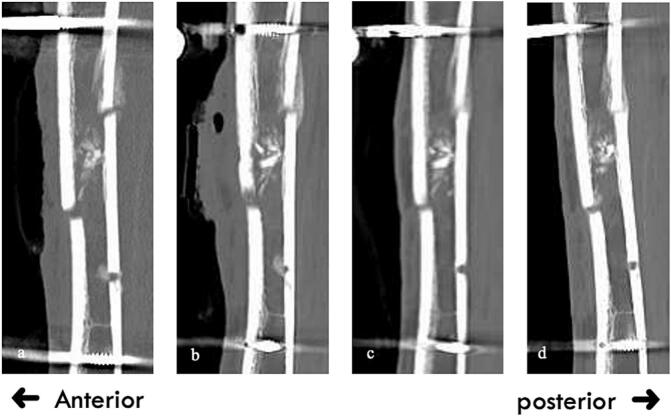
Sequential sagittal computed tomography images showing the healing process. a: 2 months after surgery. b: 3 months after surgery. c: 4 months after surgery. d: 5 months after surgery.

**Fig. 8:**
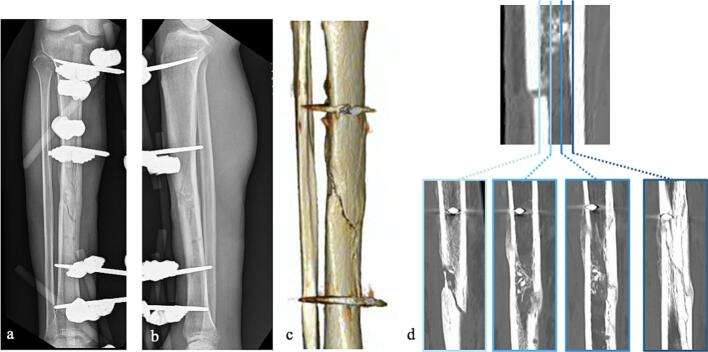
Radiological findings 5 months after surgery. a: Anteroposterior plain radiograph. b: Lateral plain radiograph. c: Three-dimensional computed tomography reconstruction. d: Axial computed tomography image.

**Fig. 9:**
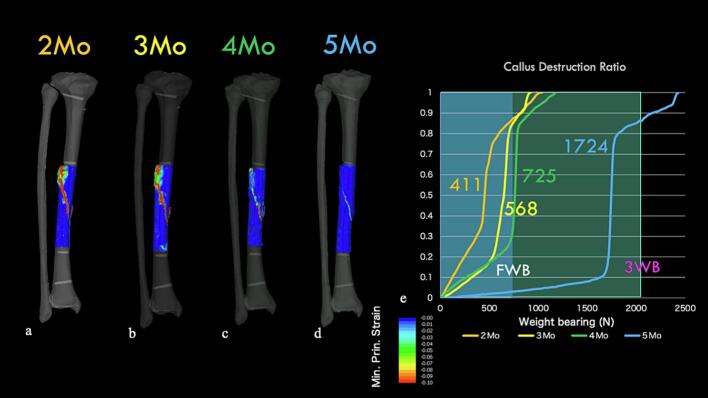
Minimum principal strain contour maps in the removal model under full weight bearing. a: 2 months after surgery: Minimum principal strain exceeding −0.1 in most of the callus regions with evident fracture displacement. b: 3 months after surgery: A decrease in fracture displacement. c: 4 months after surgery: Minimum principal strain exceeding −0.1 in some regions, suggesting potential callus failure, though displacement is minimal. d: 5 months after surgery: Strain limited to approximately −0.02 with no evidence of callus failure. e: Callus failure rate: Rapid increase in callus failure under full weight-bearing conditions up to 4 months after surgery.

Fig. 7 shows the progression of sagittal CT images. At 3 months postsurgery, although posterior bone bridging was complete, anterior bridging remained insufficient. At 5 months, anterior callus formation was observed, albeit incomplete (Fig. 8). Fig. 9 presents the minimum principal strain contours under full weight-bearing conditions in the removal model across each time point. At 2 months, the minimum principal strain fell below −0.1 in most of the callus region with displacement at the fracture site. At 3 months, the fracture displacement decreased, whereas by 4 months, concerns about callus failure emerged despite minimal displacement. However, increased displacement at the fracture site was observed at 725 N, indicating insufficient strength to permit fixator removal. At 5 months, the minimum principal strain was limited to approximately −0.02, with no callus failure having been observed up to 1724 N. Although external fixator removal was considered 6 months after surgery, the patient requested high-intensity training to allow resumption of work as a medical corpsman while wearing the external fixator. Therefore, to ensure safety, external fixator removal was performed 7 months after surgery. After removal, the patient performed his daily activities without complications and was able to resume his original duties 12 months after surgery.

## Discussion

Traditionally, decisions regarding postoperative weight-bearing protocols and timing of external fixator removal during fracture treatment have been based on physicians' subjective assessments of radiographic and clinical findings. This approach has often caused extended periods of postoperative immobilization and prolonged external fixation while prioritizing safety considerations. Conversely, attempts to expedite return to activities have occasionally promoted nonunion or refractures following fixator removal. Hence, establishing objective methods for evaluating fracture stability remains a crucial challenge in trauma care.

The development of user-friendly software developed specifically for bone FEA such as Mechanical Finder (Medical Device Approval Number: 22800BZX00023000 in Japan), has enabled orthopaedic surgeons to rapidly construct clinically relevant models. Furthermore, advances in computational performance have facilitated reductions in processing times for patient-specific CT-based finite element analyses. Considering that patient-specific CT-based FEA could be completed within 24 h of CT imaging, its results can be directly incorporated into patient care decisions.

Patient-specific CT-based FEA during external fixation revealed that full weight bearing within the first 3 months after surgery increased the risk of callus failure, prompting us to limit weight bearing to 50 % of full body weight. By the 4th month, however, our analysis predicted a low risk of callus failure under full weight-bearing conditions, allowing us to safely permit complete weight-bearing and unrestricted activities of daily living. This finding suggests that patient-specific CT-based FEA may be used to effectively determine appropriate weight-bearing protocols. Furthermore, FEA of the fixator removal model indicated sufficient recovery to permit full weight bearing 5 months after injury. However, considering that running can generate forces two to three times one's body weight, we determined that activities beyond walking would be inadvisable at the 5-month stage. Respecting the patient's desire to undergo adequate training before returning to active duty as a combat medic, we delayed fixator removal by 2 months and removed it at 7 months after injury. The ability to objectively evaluate mechanical behavior enabled us to prescribe more specific activity restrictions to the patient, which have been proven to be remarkably beneficial in clinical practice.

As the basis for the creation of a three-dimensional model, medical radiation exposure is inevitable in CT imaging. However, current scientific evidence suggests no demonstrable increase in cancer risk from low-dose (below 100 mSv) radiation exposure [6]. Moreover, effective doses from were markedly lower for peripheral CT examinations (elbow: 0.14 mSv, wrist: 0.03 mSv, knee: 0.16 mSv, and ankle: 0.07 mSv) than for torso CT scans (chest: 5.27 mSv, abdomen: 4.95 mSv, and pelvis: 4.85 mSv) [7]. Furthermore, fewer radiosensitive organs are present in the extremities than in the torso, suggesting a lower potential for adverse health effects [8]. Therefore, the clinical benefits of radiation exposure seemingly outweigh its disadvantages. Nevertheless, adherence to radiation dose optimization principles remains essential in clinical practice.

Second, patient-specific CT-based FEA remains a simulation with predictive limitations. The analysis only considers simple loading conditions and does not account for torsional or bending forces. Additionally, dynamic analysis was performed using static analysis methods. Hence, the accuracy of this approach in evaluating callus failure under impact loading conditions remains unclear.

Third, given that this study included only a single case, validating the accuracy of our evaluations was difficult. We acknowledge that this research should be considered a pilot study. Nevertheless, our work represents significant progress from traditional methods, where postoperative protocols and timing of external fixator removal were determined solely based on physician intuition and experience. Hence, the ability to quantify these parameters marks a substantial advancement. Future clinical applications across broader patient populations will be essential to validate the accuracy of this approach.

Finally, since this study was applied to an open fracture, there may be concerns about its applicability to common closed fractures. However, we are confident that this methodology should be particularly utilized for open fractures where bone healing is unstable and it is difficult to determine whether sufficient bone strength has been achieved, as the fundamental biomechanical principles of evaluating bone strength from Hounsfield values remain consistent regardless of fracture type. Previously, we have demonstrated the effectiveness of this approach in closed femoral shaft fractures treated with intramedullary nailing [9]. However, that study was based on retrospective analysis rather than prospective clinical application. The present study demonstrates the potential for prospective clinical application of this methodology.

## CRediT authorship contribution statement

**Yusuke Matsuura:** Writing – original draft, Software, Project administration, Data curation. **Takahiro Yamazaki:** Data curation. **Seiji Ohtori:** Supervision.

## Declaration of competing interest

None.

## Appendix A Supplementary data

Video 2 Video 2

Supplementary material Image 1
