# Supplementary material for: Quantitative assessment of fracture healing using patient-specific CT finite element analysis: A novel approach to external fixation management
Source: Trauma Case Rep. 2025 Aug 22;60:101245. doi: 10.1016/j.tcr.2025.101245 (PMC12455202; doi:10.1016/j.tcr.2025.101245)
Supplement: Supplementary file 3 — Supplementary material [file mmc3.pdf]

プログラム 1　疾病診断用プログラム

管理医療機器　骨強度分析プログラム　JMDN コード：71041002

骨強度評価ソフトウェア　MECHANICAL FINDER

【形状・構造及び原理等】

本プログラムは、汎用ワークステーションにインストールして使用するプログラムである。
X 線コンピュータ断層撮影装置（CT）で得られた画像を元に、更に処理され、画像部位における骨塩量を求め、診断補助のために提供するプログラムである。
患者個々の骨強度を力学的に解析する有限要素法解析機能がある。

機能

項目	仕様
CT データから直接形状抽出	CT 原画像データ上で直接解析対象となる領域をなぞる、あるいは閾値処理をほどこして半自動的に関心領域を抽出することで、忠実な骨形状抽出を行う。
オートメッシュジェネレータ	インプラント挿入した形状（EE）や ROI 抽出した CT データに対し三次元メッシュを自動生成する。
骨強度評価用解析	骨全体を三次元構造物として、有限要素法による構造解析を行い、骨密度や骨強度を評価する。<br>推奨骨塩定量ファントム：京都科学製 B-MAS200 型,QRM 製 QRM-BDC
	1) 弾性解析<br>ひび割れ、塑性を考慮しない弾性解析を行う。
	2) 非線形骨折線予測解析<br>増分法による非線形解析を行う。増分応力が加算される事によって引張強度を超えるとひび割れが生じ、それまで保持していた応力は解放され、力の釣り合いを満たすまで反復計算を行う。また、圧縮域の降伏および圧壊も考慮する。
入出力機能	解析結果を三次元画像として、数値と共に、三次元カラー表示を行う。

付帯機能

項目	機能説明
画像処理機能	1) WL/WW 変更処理
	2) Zoom 処理
	3) Pan 処理
画像計測機能	1) 距離計測
	2) 面積計測
	3) 角度計測
	4) データ値計測
	5) 画素値計測
	6) 統計処理
	7) ROI 計測
データ管理機能	データの変更、削除、移動の管理をする。

提供形態

CD-ROM / USB メモリスティック / ダウンロード

作動・動作原理

骨強度評価解析プログラムの動作原理は以下の通りである。プログラムの処理のフローも示す。

有限要素法：変位法
・弾性解析
・非線形骨折線予測解析

骨強度三次元解析
1) DICOM データを本ソフトウェア形式ファイルへ変換する。また、画像ファイル（BMP・JPEG）からの変換も行える。
2) CT ボリュームの有効範囲を制限する。
3) CT 原画像データから解析対象となる骨を抽出する。抽出された情報は解析対象となる部分：真、解析対象外の部分：偽により表現した二値化データとして保存される。
4) インプラント挿入した形状や ROI 抽出した CT データに対し３次元メッシュを自動生成する。
5) 解析材料・骨軸・解析座標の設定を行う。
6) ヤング率や降伏応力値等の材料を設定する。
7) 荷重・拘束条件などを設定する。
8) 設定された条件で解析を行い、結果を表示する。
9) メッシュ生成された形状の外側形状（シェル要素）部分を STL フォーマット、DXF フォーマットとしてファイル出力する。
10) 各機能プロセスに合った表示機能を備え、表示画面を画像ファイルや動画ファイルとして保存する事も可能。また、アニメーション表示や VRML 出力も備えている。
11) グラフ機能 材料特性・解析結果を用いてのグラフ描画が行える。

【使用目的又は効果】

画像診断装置等から提供された人体の画像情報をコンピュータ処理し、骨強度の計算を行う。処理後の画像情報を診断補助のために提供する。

【使用方法等】

画像診断装置等から提供された人体の画像情報をコンピュータ処理し、骨強度の計算を行う。処理後の画像情報を診断補助のために提供する。

汎用ワークステーションの要件

本品目は、下記の仕様を満たす汎用 IT 機器に製造販売業者が指定した方法（添付文書又はプログラムに含めた電磁的記録に記載された手順）でインストールして使用する。
汎用 IT 機器は、患者環境外に設置する。

汎用 IT 機器の仕様

・ハードウェア環境

OS	推奨<br>日本語／英語 Windows8　64 ビット<br>日本語／英語 Windows10　64 ビット<br>日本語／英語 Windows11
プロセッサ	推奨 Intel 64 準拠
メモリ	推奨 8GB 以上

・ハードウェアレンダリング

グラフィックス	・フルカラーグラフィックスボード<br>・推奨サイズ 1280×1024 以上<br>・OpenGL 対応グラフィックスボード<br>・Nvidia 製グラフィックスボード(推奨)

取扱説明書を必ずご参照ください。

使用方法

1) 使用準備
   (1) インストール先の機器の電源を入れる。
   (2) 本プログラムを起動する。
2) 操作
   (1) 画像データを取得する。
   (2) 機能を選択する。
   (3) 三次元画像表示等を行う。
   (4) 結果を保存する。
3) 終了
   (1) 画面上の終了アイコンをクリックするか、メニュー項目から終了機能を選択し本プログラムを終了させる。
   (2) 必要に応じて電源を切る。

操作方法の詳細については、本プログラムの取扱説明書を参照すること。

推奨CT撮影条件

・管電圧：120kV程度
・ガントリチルト：0°
・スライス間隔：2mm以下
・画素間隔：1mm以下

【使用上の注意】

使用注意

1) ネットワークに接続した環境で使用する場合は、コンピュータウイルス及び情報の漏洩等に注意すること。
2) プログラムの同時使用に注意すること。

重要な基本的注意

1) 推奨仕様を満たす機器にインストールすること
2) 本品の解析結果を根拠に診断を確定するのではなく、あくまで診断の補助にのみ使用すること。
3) CT 画像上に金属アーチファクトがある場合、術者が解析結果に対してハレーションの影響を評価可能と判断した場合のみ使用すること。
4) 本品は、必ずネットワークに接続されていない機器で解析を行うこと。ネットワークに接続した場合、サイバーセキュリティを確保出来ず、患者情報が漏えいする恐れ場ある。
5) ワークステーションのパスワード管理を徹底し、個人情報を削除する機能により、院外へ持ち出す際は必ず匿名化すること。
6) 本ソフトウェアには、解析する際の手法として「非線形骨折線予測解析」という項目があり、この「骨折線」は、「臨床的な骨折」を指すものではなく、あくまで解析モデル上の「破壊」現象を表現しているもので、臨床上の「骨折」を予測する上で、解析モデル上の「破壊」をどのように結びつけるかは使用者が定義する必要がある。
7) 本品の解析結果は、使用者が設定した解析条件次第で変わるため、設定した解析条件の妥当性については、使用者が各々実試験の結果を踏まえて確認すること。

【保守・点検に係る事項】

使用者による保守点検事項

自己点検プログラムが起動する場合は、問題がなきことを確認してから、使用すること。

【主要文献及び文献請求先】

文献請求先

株式会社計算力学研究センター
東京都品川区戸越1-7-1 東急戸越ビル
電話番号 03-3785-3033
Fax番号 03-3785-6066

【製造販売業者及び製造業者の氏名又は名称等】

製造販売業者

株式会社計算力学研究センター
電話番号 03-3785-3033

製造業者

株式会社計算力学研究センター
電話番号 03-3785-3033

連絡先

株式会社計算力学研究センター
電話番号 03-3785-3033

取扱説明書を必ずご参照ください。
